# Hospitals' collection and use of data to address social needs and social determinants of health

**DOI:** 10.1111/1475-6773.14341

**Published:** 2024-07-02

**Authors:** Chelsea Richwine, Samantha Meklir

**Affiliations:** ^1^ Office of the National Coordinator for Health Information Technology U.S. Department of Health and Human Services Washington DC USA

**Keywords:** health‐related social needs, population health, public health, screening, social determinants of health

## Abstract

**Objective:**

To assess differences in hospitals' collection and use of data on patients' health‐related social needs (HRSN) by availability of programs or strategies in place to address patients' HRSN and social determinants of health (SDOH) of communities.

**Data Sources:**

The 2021 American Hospital Association Annual Survey and 2022 Information Technology (IT) Supplement.

**Study Design:**

This cross‐sectional study described hospitals' engagement in screening and the availability of programs or strategies to address nine different HRSN. We assessed differences in screening rates and uses of data collected through screening among hospitals with and without programs or strategies in place to address HRSN or SDOH using Chi‐squared tests of independence.

**Data Collection/Extraction Methods:**

Analyses were restricted to IT Supplement respondents with complete data for social needs questions asked in the Annual Survey (*N* = 1997).

**Principal Findings:**

In 2022, hospitals used social needs data collected through screening for various purposes including discharge planning and clinical decision‐making at their hospital as well as to refer patients to needed resources and assess community‐level needs. Hospitals with a program or strategy in place had higher rates of screening across all domains and higher rates of using of data collected through screening for uses involving exchange or coordination with external entities.

**Conclusions:**

Collection of social needs data may help inform the development of programs or strategies to address HRSN and SDOH, which in turn can enable providers to screen for these needs and use the data in the near term for care delivery and in the long term to address community and population needs.

**What is known on this topic**

- Most hospitals screen for patients' health‐related social needs (HRSN), but screening rates vary by domain.
- Many hospitals have programs or strategies to address HRSN or community social determinants of health (SDOH), but it is unclear how these relate to use of data captured through screening.

**What this study adds**

- Some of the most screened for HRSN had substantially lower rates of programs or strategies to address identified needs, leaving hospitals potentially ill‐equipped to effectively use data captured through screening.
- Hospitals use data collected through screening for various purposes, including internal uses (e.g., clinical decision‐making, discharge planning) as well as external uses (e.g., referrals, community needs assessments, population health analytics).
- Having programs or strategies to address HRSN and SDOH is associated with higher rates of screening and use of data for referrals, population health analytics, and assessing community needs.

## 1 INTRODUCTION

Health‐related social needs (HRSN) refer to individuals' social needs (e.g., access to healthy food, reliable transportation) that affect health and well‐being and often stem from broader conditions of the environment that govern and shape the communities in which people live.^1^, ^2^ In order to develop programs and strategies that adequately address the needs of individuals and communities, greater attention has been devoted to distinguishing *social determinants of health* (SDOH)—conditions of the environment that can exert a positive or negative influence on health—from individual HRSN that, left unmet, may adversely affect an individual's health.^1^, ^3^ Identifying HRSN is a critical first step toward enabling healthcare providers, including those in inpatient settings, to refer patients to resources or social services that meet their unique needs and to inform the development of programs and strategies to address HRSN of patients and SDOH of communities.^4^, ^5^

While many programs and strategies claim to address SDOH, most of these efforts are aimed at identifying and meeting the HRSN of individuals rather than addressing inequities in the SDOH of communities.^3^ Recent studies have shown that most hospitals screen for HRSN in some capacity.^6^, ^7^, ^8^, ^9^, ^10^, ^11^ As of 2022, 83% of nonfederal acute care hospitals reported collecting data on patients' HRSN, of which 74% used a structured electronic screening tool consisting of either an externally established tool, a customized/home grown tool, or a combination of both, and 29% used diagnosis codes.^7^ Further, most hospitals have at least some dedicated efforts in place to address HRSN or SDOH such as screening, assisting with transitions of care, and making connections to social service organizations; however, efforts to assist patients who screen positive for HRSN are often fragmented and external partnerships to address HRSN vary by hospital characteristics.^6^, ^9^, ^10^, ^11^ In a 2017 survey of US hospitals, 72% of respondents reported a lack of dedicated funds to address HRSN and about 40% reported a lack of capabilities to measure the outcomes of their activities.^6^ In a 2022 survey of health information professionals with roles in health information data collection and management, nearly half of respondents (41%) cited an inability to address HRSN (e.g., due to lack of expertise or resources) as a challenge to social needs data collection. A lack of trained workforce or of organizational policy on screening (93% and 87%, respectively) were other major barriers to HRSN data collection.^8^ Communication issues between health and social service sectors also posed barriers to using data to make patient referrals and to identify and assess community‐level needs.^8^

Taken together, the existing literature suggests that while social needs screening and efforts to address these needs are prevalent at US hospitals, there is often a disconnect between the collection and use of data captured through screening. It is unclear whether hospitals use data internally to support patient care or for uses involving entities external to the hospital, or how the availability of programs relates to data collection and subsequent use. Our study contributes to the literature by leveraging data from two nationally representative surveys of hospitals in 2022 to describe programs or strategies in place for addressing HRSN at US hospitals and how the availability of these programs or strategies relates to the collection and use of data captured through screening. We assess differences in the collection and use of data among hospitals with and without programs or strategies in place, with a focus on screening for nine different HRSN and six specific uses of the data centered around internal uses for inpatient care delivery (i.e., discharge planning, clinical decision‐making) as well as uses involving external entities (i.e., making referrals to social service organizations, informing community needs assessments or other equity initiatives, population health analytics) and quality management.

## 2 DATA AND METHODS

Data come from the 2021 American Hospital Association Annual Survey and 2022 Information Technology (IT) Supplement to the Annual Survey. The 2021 Annual Survey was fielded March to August 2022 and had a total of 6201 respondents (72% response rate). The 2022 IT Supplement was fielded July to December 2022 and had a total of 3127 respondents (50% response rate). Analyses were restricted to IT Supplement respondents with complete data for the social needs questions asked in the Annual Survey (*N* = 1997). A logistic regression model was used to predict the propensity of survey response as a function of hospital characteristics, including size, ownership, teaching status, system membership, and availability of a cardiac intensive care unit, urban status, and region. Hospital‐level weights were derived by the inverse of the predicted propensity.

The Annual Survey asks hospitals if they have programs or strategies to address nine specific HRSN of patients or SDOH of communities and whether they screen patients for social needs (Appendix A). In this study, we described hospitals' engagement in screening for nine HRSN and the availability of programs or strategies for addressing HRSN and SDOH related to the following domains: education, employment and income, food insecurity and hunger, health behaviors, housing, interpersonal violence, social isolation, transportation, and utility needs. While health behaviors are often viewed as distinct from HRSN, we included them here since health behaviors reflect not only individual choice but are influenced by societal organization and structural inequalities.^12^ The IT Supplement asks about hospitals' collection and use of data on patients' HRSN for six different purposes including internal uses for inpatient care delivery (i.e., discharge planning, clinical decision‐making), uses involving external entities (i.e., making referrals to social service organizations, informing community needs assessments or other equity initiatives, population health analytics), and quality management (Appendix B). We assessed differences in screening rates and uses of data collected through screening among hospitals with and without programs or strategies in place to address HRSN or SDOH using Chi‐squared tests of independence. This study did not require approval or consent from an ethics committee because it did not involve human participants.

## 3 RESULTS

**FIGURE 1:**
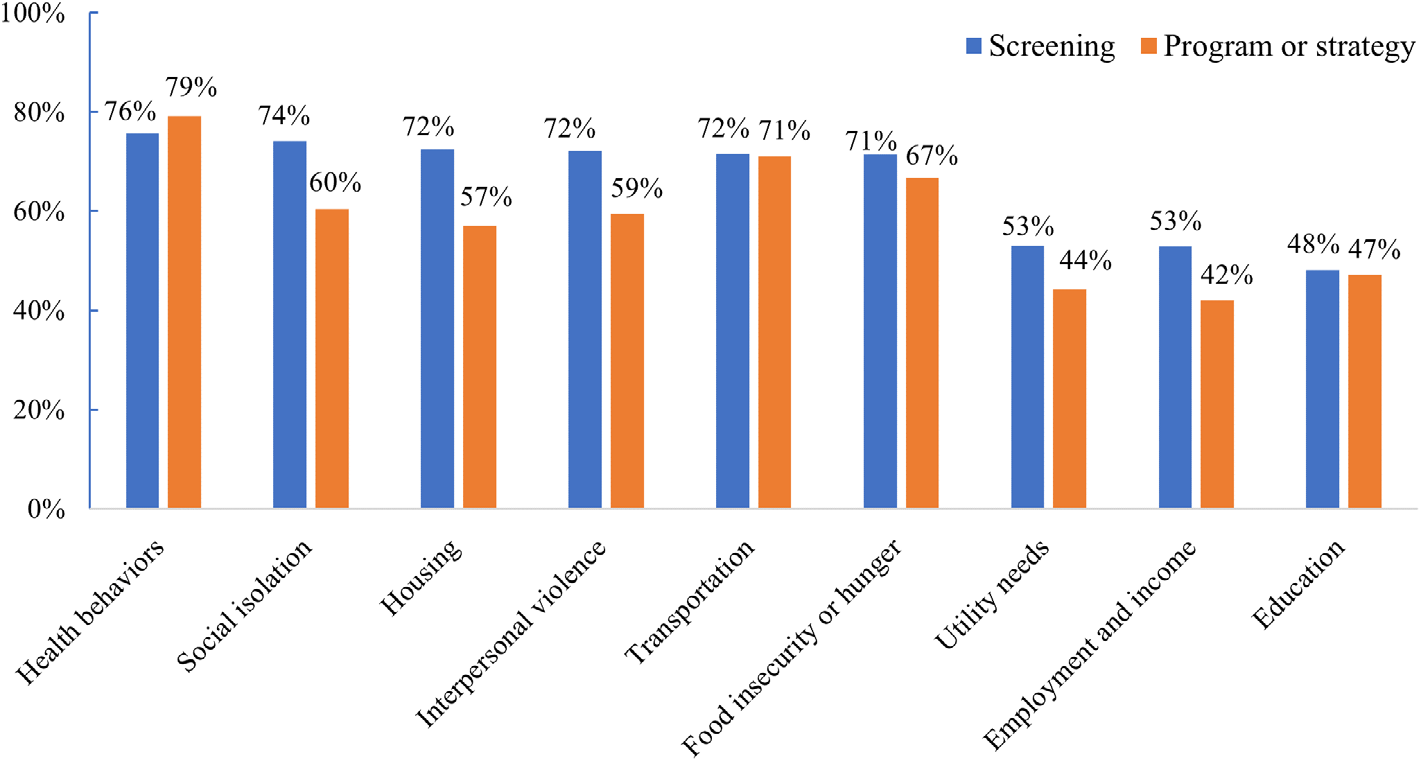
Rates of screening and availability of programs or strategies for addressing social needs at US hospitals in 2022. Denominator includes respondents who answered questions about screening and availability of programs/strategies for addressing social needs (*N* = 1997). *Source*: 2021 American Hospital Association Annual Survey.

In 2022, rates of hospital screening and availability of programs or strategies for addressing HRSN or SDOH varied by domain (Figure 1). Screening rates were the highest for health behaviors (76%), social isolation (74%), housing, interpersonal violence, and transportation (72%, respectively), and food insecurity or hunger (71%). Rates were the lowest for utility needs and employment and income (53%, respectively) and education level screening (48%). The availability of programs or strategies to address HRSN or SDOH was the highest for health behaviors (79%), transportation (71%), and food insecurity (67%) and the lowest for education (47%), utility needs (44%), and employment and income (42%). Across several domains, there was a gap between rates of screening for a specific HRSN and availability of programs or strategies to address that need. For instance, 74% of hospitals screened for social isolation in 2022 but only 60% had a program or strategy to address this need.

For each of the nine domains assessed in the AHA Annual Survey, hospitals with a program or strategy to address a specific HRSN or SDOH had significantly higher rates of screening for the respective HRSN compared to hospitals without a program or strategy in place (Table 1, Column 1). For example, 92% of hospitals with a housing‐related program or strategy in place screened for housing‐related social needs compared to just 47% of hospitals that did not have a housing‐related program or strategy. Overall screening rates for any type of HRSN (80% nationally) assessed in the IT Supplement were also significantly higher among hospitals with programs or strategies to address HRSN or SDOH at their hospital (Table 1, Column 2). For example, 87% of hospitals with a food insecurity or hunger‐related program or strategy in place screened for any HRSN compared to 65% of hospitals that did not have a food insecurity or hunger‐related program or strategy.

**TABLE 1:** Social needs screening among hospitals, by availability of programs or strategies to address health‐related social needs and social determinants of health.

	Data collection	
Program or strategy	(1) Screen for specific HRSN^a^	(2) Any HRSN screening (80%)^b^
Housing		
Yes (*N* = 1276)	92%*	86%*
No (*N* = 721)	47%	71%
Food insecurity or hunger		
Yes (*N* = 1524)	89%*	87%*
No (*N* = 473)	37%	65%
Utility needs		
Yes (*N* = 1017)	86%*	87%*
No (*N* = 980)	27%	74%
Interpersonal violence		
Yes (*N* = 1310)	91%*	86%*
No (*N* = 687)	44%	71%
Transportation		
Yes (*N* = 1554)	86%*	84%*
No (*N* = 443)	36%	69%
Employment and income		
Yes (*N* = 986)	79%*	89%*
No (*N* = 1011)	34%	73%
Education		
Yes (*N* = 1059)	75%*	88%*
No (*N* = 938)	25%	72%
Social isolation		
Yes (*N* = 1297)	90%*	83%*
No (*N* = 700)	50%	74%
Health behaviors		
Yes (*N* = 1691)	85%*	82%*
No (*N* = 306)	41%	71%

*Note*: Denominator includes all respondents who answered questions about screening and availability of programs or strategies for addressing health‐related social needs (HRSN) or social determinants of health (SDOH) (*N* = 1997).

*Source*: 2021 American Hospital Association Annual Survey and 2022 IT Supplement to the Annual Survey.

^a^ Indicates the share of hospitals that reported screening for specific HRSN referenced in the “Program or strategy” column in the AHA Annual Survey.

^b^ Indicates the share of hospitals that reported collecting any data on individual patients' HRSN in the AHA IT Supplement.

* Indicates a statistically significant difference in screening by availability of program or strategy as assessed by a Chi‐squared test of independence (*p* < 0.05). Test statistics available in Appendix C.

Among hospitals that collected social needs data through screening (*N* = 1691, 80%), the most common uses of data collected included discharge planning (88%), making referrals to social service organizations (83%), and clinical decision‐making (80%). Hospitals also used the data to inform community needs assessments or other equity initiatives (58%), to conduct population health analytics (53%), and quality management (51%) (Table 2). Hospitals' use of data collected through screening to inform discharge planning or clinical decision‐making did not differ significantly by availability of programs or strategies (Table 2, Columns 1–2). In contrast, hospitals that screened for HRSN and had a program or strategy to address specific HRSN used data for making referrals, informing community needs assessments, population health analytics, and quality management at higher rates compared to hospitals that screened for HRSN but did not have a program or strategy in place (Table 2, Columns 3–6). For example, 60% of hospitals with a transportation‐related program or strategy in place used data collected through screening to inform community needs assessments compared to 51% of hospitals that screened for HRSN but did not have a transportation‐related program or strategy.

**TABLE 2:** Uses of data collected by hospitals engaged in screening, by availability of programs or strategies to address health‐related social needs and social determinants of health.

	Data use					
Program or strategy	(1) Discharge planning (88%)	(2) Clinical decision making (80%)	(3) Making referrals (83%)	(4) Community needs assessment (58%)	(5) Population health analytics (53%)	(6) Quality management (51%)
Housing						
Yes (*N* = 1127)	88%	78%	84%	62%*	60%*	52%
No (*N* = 564)	89%	84%*	81%	51%	41%	48%
Food insecurity or hunger						
Yes (*N* = 1354)	88%	79%	85%*	61%*	62%*	53%*
No (*N* = 337)	89%	83%	78%	48%	29%	44%
Utility needs						
Yes (*N* = 905)	88%	81%	88%*	67%*	65%*	58%*
No (*N* = 786)	89%	80%	79%	49%	41%	44%
Interpersonal violence						
Yes (*N* = 1158)	87%	79%	86%*	62%*	60%*	54%*
No (*N* = 533)	90%	82%	78%	50%	40%	44%
Transportation						
Yes (*N* = 1347)	88%	80%	85%*	60%*	57%*	54%*
No (*N* = 344)	88%	80%	76%	51%	40%	42%
Employment and income						
Yes (*N* = 892)	89%	82%	86%*	66%*	62%*	55%*
No (*N* = 799)	88%	79%	80%	50%	45%	47%
Education						
Yes (*N* = 950)	87%	82%	84%	66%*	61%*	58%*
No (*N* = 741)	90%	79%	82%	49%	43%	43%
Social isolation						
Yes (*N* = 1127)	88%	80%	84%	59%	57%*	52%
No (*N* = 564)	89%	82%	81%	55%	46%	48%
Health behaviors						
Yes (*N* = 1457)	88%	79%	84%	60%*	57%*	52%
No (*N* = 234)	92%	86%	78%	48%	35%	43%

*Note*: Denominator only includes hospitals that reported collecting any data on individual patients' health‐related social needs in the AHA IT Supplement (*N* = 1691).

*Source*: 2021 American Hospital Association Annual Survey and 2022 IT Supplement to the Annual Survey.

* Indicates a statistically significant difference in screening by availability of program or strategy as assessed by a Chi‐squared test of independence (*p* < 0.05). Test statistics available in Appendix C.

## 4 DISCUSSION

In 2022, most hospitals screened for at least one type of HRSN, but screening rates varied by domain, ranging from 48% for education to 76% for health behaviors. Variation in screening may be due, in part, to different tools used for social needs screening which often include specific domains recommended for capture in electronic health record systems based on utility and readiness of available measures in each domain.^7^, ^13^ Similarly, most hospitals had programs or strategies in place to address HRSN, ranging from 42% for employment and income to 69% for health behaviors. While prior work has noted the cooccurrence between screening and programs or strategies to address specific HRSN,^11^ some of the most screened for HRSN—social isolation, housing, and interpersonal violence—had substantially lower rates of programs or strategies to address these needs, which suggests a lack of capacity to support social interventions in communities, leaving hospitals ill‐equipped to effectively address certain needs identified through screening.

Hospitals with a program or strategy in place to address HRSN or SDOH had significantly higher rates of screening for specific HRSN and engagement in any type of HRSN screening compared to hospitals without dedicated efforts to address the identified needs of patients or communities. These findings, together with existing literature on barriers to addressing the needs of patients and communities, suggest that the collection of social needs data may help inform the development of programs or strategies to address HRSN or SDOH—such as building partnerships with community and social service organizations—which in turn can enable providers to screen for these needs and ensure the data are available to use for different purposes. These findings provide a helpful baseline for hospitals' engagement in HRSN screening in 2022 before new screening requirements for hospitals participating in the Centers for Medicare & Medicaid Services' Inpatient Quality Reporting program became voluntary in 2023 and required in 2024, which is important for understanding potential approaches and challenges for hospitals and policymakers as they transition to these new screening requirements.^14^ Our findings may offer useful insights for hospital leadership to consider in their development of programs or strategies to support these new requirements. For instance, hospitals could consider new uses of screening data to support care delivery functions (as they do for clinical decision‐making and discharge planning), improve the efficiency of clinical workflows, and inform community needs assessments and other health equity initiatives. Further, given prior literature citing an inability to address HRSN (due to lack of expertise or resources) as a barrier to social needs data collection,^8^ having dedicated resources or strategies in place may alleviate providers' concerns about collecting HRSN data as the program or strategy enables them to more effectively use these data to refer patients to needed resources. Having dedicated resources or strategies and making these known to patients may also help increase patient comfort with social needs data sharing and confidence that data collected through screening can be used to help meet their needs.^15^

Hospitals that collected social needs data used the information for various purposes, with the most common being internal uses of the data to support patient care, including clinical decision‐making and discharge planning. This finding aligns with recent studies that found social needs and social risk data are increasingly being used by healthcare teams to support decision‐making at the point of care and improve patient‐centered care delivery,^16^, ^17^, ^18^ which speaks to the value of screening for hospitals as part of care delivery even if they lack programs or strategies to address patients' specific unmet HRSN.

In addition to internal uses, hospitals frequently used data collected through screening for making referrals to social service organizations. Data were also used for other purposes involving coordination or partnerships with organizations such as informing community needs assessments or other equity initiatives and population health analytics. These uses of data are important for identifying specific needs of communities or disparities in the population, which may be used to inform hospital decision‐making around dedicating resources to the development of programs or strategic partnerships. Notably, rates of use for the purposes of making referrals, informing community needs assessments, and population health analytics were higher among hospitals with programs or strategies to address specific HRSN or SDOH, indicating a relationship between the availability of resources or partnerships and the ability of hospitals to more effectively use data captured through screening for these purposes.

Increasing the collection of actionable social needs data and using such data to connect patients with social care providers supports the goals and strategic next steps identified in the U.S. Department of Health and Human Services' (HHS) Strategic Approach to Addressing Social Determinants of Health to Advance Health Equity – At a Glance as well as the U.S. Playbook to Address SDOH and the HHS Call to Action to address HRSN through community partnerships.^19^, ^20^, ^21^, ^22^ While our findings suggest data collected through screening can be used internally to support clinical decision‐making and discharge planning, without programs or strategies in place and dedicated resources to address HRSN and SDOH involving cross‐sector partnerships with sufficient service capacity, increased screening may not facilitate uses of the data aimed at improving both patient and population health outcomes. Ongoing efforts to improve cross‐sector partnerships and health and social services connections will be critical to making social needs screening actionable to address patients' HRSN and community‐level SDOH. While our findings suggest the availability of programs or strategies at hospitals to address HRSN or SDOH is linked to higher rates of data collection and use for various purposes, future qualitative work would be valuable for uncovering what these programs entail, whether programs were developed with community input, and how program activities and strategies align with health equity goals that can be linked to measurable outcomes.

Understanding how programs and involved workforce can leverage health IT enabled screening tools^23^ will also be critical to informing the various ways collected data can be used to effectively address HRSN and SDOH. As HRSN data become more readily available in hospital settings, this information could increasingly be used for purposes beyond care coordination and referrals to and from community‐based organizations (closed‐loop referrals), including broadening use of data for informing community health needs and identifying gaps in social care resources, as well as research and population health analytics. Potential reuse of HRSN data for various purposes—both within and across different health systems—speaks to the importance of capturing these data in a standardized format using validated screening instruments that would enable identification of a more complete set of HRSN and facilitate interoperable exchange across the care continuum. Finally, understanding how programs train workforce and engage in shared governance or SDOH information exchange with communities will be important for identifying future investments, approaches, and resources to address HRSN of patients and SDOH in communities.

## 5 LIMITATIONS

Our study is subject to limitations. One important limitation is reliance on a cross‐sectional, self‐reported survey to assess hospitals' collection and use of HRSN data and their awareness of the availability of programs to address HRSN of patients and SDOH in communities. Survey data are self‐reported by hospitals and thus responses reflect the knowledge and recall of the respondent rather than actual technical capabilities or programmatic activities at hospitals. Further, we are limited in our knowledge of what these programs or strategies entail and whether they were developed in partnership with community organizations. Another important limitation of this study is that we do not establish a causal relationship between the availability of programs or strategies to address HRSN or SDOH and subsequent screening activities. It is possible that screening for HRSN revealed certain patient or community needs that led to the development of programs or strategies to address those needs, or that the availability of these programs or strategies enabled providers to collect and use data for various purposes more effectively. Notwithstanding these limitations, we believe we have filled an important gap in the literature by establishing a consistent, significant relationship between the presence of dedicated efforts to address HRSN or SDOH and rates of data collection and use to meet individual and community needs.

## 6 CONCLUSION

Hospitals with a program or strategy in place to address HRSN of patients or SDOH in communities had higher rates of screening and use of data for purposes involving other organizations or partnerships, which suggests having programs or strategies to address specific HRSN or SDOH may enable providers to use data captured through screening to refer patients to needed resources in the near term and help meet community and population needs in the long term. Hospitals commonly used data collected through screening internally to support patient care, including clinical decision‐making and discharge planning, which demonstrates the value of social needs screening for hospitals in routine care delivery workflows as well as for developing strategies or forming strategic partnership to address HRSN of patients and SDOH of communities.

As we can expect increasing rates of screening activities by hospitals to take hold, future work should focus on understanding what successful programs entail and the extent to which strategies involve formal partnerships with community and social service organizations with the capacity to support social interventions. This would help inform approaches for hospitals considering such programs and assist those interested in approving their current programs or strategies. This involves, in part, understanding whether the data collected through screening are captured in a standardized format that would facilitate the capture, exchange, and use of data for different purposes including to inform and measure improvements in patient and population health, for research, as part of electronic decision support or care planning, and for quality measurement development.

## Supporting information


**Appendix S1.** Supporting information.
