# Supplementary material for: Hospitals' collection and use of data to address social needs and social determinants of health
Source: Health Serv Res. 2024 Jul 2;59(6):e14341. doi: 10.1111/1475-6773.14341 (PMC11622261; doi:10.1111/1475-6773.14341)
Supplement: Supplementary file 1 — Appendix S1. Supporting information. [file HESR-59-0-s001.docx]

**Appendix**

**Appendix A. Survey Questions from AHA Annual Survey**

1. Which social needs of patients/social determinants of health in communities does your hospital or health system have programs or strategies to address? (Check all that apply)

a.  Housing (instability, quality, financing)

b.  Food insecurity or hunger

c.  Utility needs

d.  Interpersonal violence

e.  Transportation

f.  Employment and income

g.  Education

h.  Social isolation (lack of family and social support)

i.  Health behaviors

j.  Other, please describe: ________________________________________

1. Does your hospital or health system screen patients for social needs?

 Yes, for all patients  Yes, for some patients  No (skip to question 3)

2a. If yes, please indicate which social needs are assessed. (Check all that apply)

1.  Housing (instability, quality, financing)

2.  Food insecurity or hunger

3.  Utility needs

4.  Interpersonal violence

5.  Transportation

6.  Employment and income

7.  Education

8.  Social isolation (lack of family and social support)

9.  Health behaviors

10.  Other, please describe: ________________________________________

**Appendix B. Survey Questions from AHA IT Supplement**

**3.** Does your hospital routinely collect data on individual patients' health related social needs (often referred to as social determinants of health) such as transportation, housing, food insecurity or other?

### a.  Yes, routinely b.  Yes, but not routinely c.  No (go to 6)

### d.  Don’t know (go to 6)

4a. How are data on individual patient’s health related social needs recorded at your hospital? (Check all that apply)

### 1. Structured electronic screening tool (including ‘keying in’ information from a paper form) (go to 4b)

2. Free text note (go to 5)

3. Diagnosis codes (e.g., ICD-10-CM Z codes) (go to 5)

### 4. Non-electronic methods (including those that are scanned into the EHR) (go to 5)

**5.** How does your hospital use data on patients' health related social needs documented at your hospital? **(**Check all that apply)

### a. For referrals to social service organizations

### b. For population health analytics

### c. For quality management

### d. To inform community needs assessment or other equity initiatives

e. To inform clinical decision making

### f. To inform discharge planning

### g. Other, please list:

**Appendix C.**

**Table 1.** Social needs screening among hospitals, by availability of programs or strategies to address HRSN and SDOH

| Program / Strategy | (1)  Screen for specific HRSN^a^ | | (2)  Any HRSN screening (80%) | |
| --- | --- | --- | --- | --- |
| Housing | % | χ^2^ | % | χ^2^ |
| Yes (N=1,276) | **92%** | 483.31 | **86%** | 68.93 |
| No (N=721) | 47% | 0.000 | 71% | 0.000 |
| Food insecurity or hunger |  |  |  |  |
| Yes (N= 1,524) | **89%** | 582.17 | **87%** | 126.34 |
| No (N=473) | 37% | 0.000 | 65% | 0.000 |
| Utility needs |  |  |  |  |
| Yes (N= 1,017) | **86%** | 680.28 | **87%** | 53.94 |
| No (N=980) | 27% | 0.000 | 74% | 0.000 |
| Interpersonal violence |  |  |  |  |
| Yes (N= 1,310) | **91%** | 532.86 | **86%** | 63.83 |
| No (N=687) | 44% | 0.000 | 71% | 0.000 |
| Transportation |  |  |  |  |
| Yes (N=1,554) | **86%** | 497.71 | **84%** | 55.96 |
| No (N=443) | 36% | 0.000 | 69% | 0.000 |
| Employment and income |  |  |  |  |
| Yes (N=986) | **79%** | 411.39 | **89%** | 80.43 |
| No (N= 1,011) | 34% | 0.000 | 73% | 0.000 |
| Education |  |  |  |  |
| Yes (N=1,059) | **75%** | 501.55 | **88%** | 70.20 |
| No (N=938) | 25% | 0.000 | 72% | 0.000 |
| Social isolation |  |  |  |  |
| Yes (N=1,297) | **90%** | 384.11 | **83%** | 26.14 |
| No (N=700) | 50% | 0.000 | 74% | 0.000 |
| Health behaviors |  |  |  |  |
| Yes (N=1,691) | **85%** | 339.95 | **82%** | 25.82 |
| No (N=306) | 41% | 0.000 | 71% | 0.001 |

**Source**: 2021 American Hospital Association Annual Survey and 2022 IT Supplement to the Annual Survey.

**Notes**: Denominator includes all respondents who answered questions about screening and availability of programs or strategies for addressing social needs (*N* = 1,997). ^a^Indicates the share of hospitals who reported screening for specific HRSNs referenced in the “Program or strategy” column in the AHA Annual Survey. ^b^Indicates the share of hospitals who reported collecting any data on individual patients’ HRSNs in the AHA IT Supplement. % = Weighted percent, χ^2^ = Chi-squared test-statistic with *P*-value reported below.

**Table 2.** Uses of data collected by hospitals engaged in screening, by availability of programs or strategies to address HRSN and SDOH

| Program/Strategy | (1)  Discharge planning (88%) | | (2)  Clinical decision-making (80%) | | (3)  Making referrals (83%) | | (4)  Community needs assessment (58%) | | (5)  Population health analytics (53%) | | (6)  Quality management (51%) | |
| --- | --- | --- | --- | --- | --- | --- | --- | --- | --- | --- | --- | --- |
|  |  |  |  |  |  |  |  |  |  |  |  |  |
| Housing | % | χ^2^ | % | χ^2^ | % | χ^2^ | % | χ^2^ | % | χ^2^ | % | χ^2^ |
| Yes (N=1,127) | 88% | 0.42 | 78% | 7.08 | 84% | 1.83 | **62%** | 19.90 | **60%** | 53.45 | 52% | 2.57 |
| No (N=564) | 89% | 0.564 | **84%** | 0.036 | 81% | 0.339 | 51% | 0.001 | 41% | 0.000 | 48% | 0.213 |
| Food insecurity or hunger |  |  |  |  |  |  |  |  |  |  |  |  |
| Yes (N= 1,354) | 88% | 0.20 | 79% | 3.85 | **85%** | 12.70 | **61%** | 23.81 | **62%** | 147.57 | **53%** | 11.00 |
| No (N=337) | 89% | 0.705 | 83% | 0.155 | 78% | 0.016 | 48% | 0.001 | 29% | 0.000 | 44% | 0.018 |
| Utility needs |  |  |  |  |  |  |  |  |  |  |  |  |
| Yes (N= 905) | 88% | 0.30 | 81% | 0.05 | **88%** | 24.58 | **67%** | 56.37 | **65%** | 95.15 | **58%** | 36.85 |
| No (N=786) | 89% | 0.613 | 80% | 0.858 | 79% | 0.000 | 49% | 0.000 | 41% | 0.000 | 44% | 0.000 |
| Interpersonal violence |  |  |  |  |  |  |  |  |  |  |  |  |
| Yes (N= 1,158) | 87% | 2.27 | 79% | 1.37 | **86%** | 15.34 | **62%** | 20.59 | **60%** | 60.81 | **54%** | 16.56 |
| No (N=533) | 90% | 0.179 | 82% | 0.344 | 78% | 0.005 | 50% | 0.001 | 40% | 0.000 | 44% | 0.001 |
| Transportation |  |  |  |  |  |  |  |  |  |  |  |  |
| Yes (N=1,347) | 88% | 0.03 | 80% | 0.00 | **85%** | 18.46 | **60%** | 8.97 | **57%** | 34.15 | **54%** | 18.43 |
| No (N=344) | 88% | 0.879 | 80% | 0.967 | 76% | 0.003 | 51% | 0.031 | 40% | 0.000 | 42% | 0.002 |
| Employment and income |  |  |  |  |  |  |  |  |  |  |  |  |
| Yes (N=892) | 89% | 0.66 | 82% | 1.40 | **86%** | 9.25 | **66%** | 46.18 | **62%** | 51.03 | **55%** | 11.90 |
| No (N=799) | 88% | 0.455 | 79% | 0.319 | 80% | 0.025 | 50% | 0.000 | 45% | 0.000 | 47% | 0.005 |
| Education |  |  |  |  |  |  |  |  |  |  |  |  |
| Yes (N=950) | 87% | 2.75 | 82% | 2.20 | 84% | 2.13 | **66%** | 46.54 | **61%** | 55.65 | **58%** | 40.05 |
| No (N=741) | 90% | 0.126 | 79% | 0.221 | 82% | 0.291 | 49% | 0.000 | 43% | 0.000 | 43% | 0.000 |
| Social isolation |  |  |  |  |  |  |  |  |  |  |  |  |
| Yes (N=1,127) | 88% | 0.14 | 80% | 1.05 | 84% | 1.45 | 59% | 2.45 | **57%** | 18.83 | 52% | 3.28 |
| No (N=564) | 89% | 0.737 | 82% | 0.392 | 81% | 0.397 | 55% | 0.226 | 46% | 0.001 | 48% | 0.154 |
| Health behaviors |  |  |  |  |  |  |  |  |  |  |  |  |
| Yes (N=1,457) | 88% | 5.25 | 79% | 7.37 | 84% | 7.72 | **60%** | 14.67 | **57%** | 47.32 | 52% | 8.20 |
| No (N=234) | 92% | 0.061 | 86% | 0.070 | 78% | 0.080 | 48% | 0.007 | 35% | 0.000 | 43% | 0.043 |

**Source**: 2021 American Hospital Association Annual Survey and 2022 IT Supplement to the Annual Survey.

**Notes**: Denominator only includes hospitals that reported collecting any data on individual patients’ HRSNs in the AHA IT Supplement (*N* = 1,691). % = Weighted percent, χ^2^ = Chi-squared test-statistic with *P*-value reported below.
